# Improving exercise adherence in ankylosing spondylitis through individualized education and follow-up: a pilot randomized trial

**DOI:** 10.1007/s11845-026-04364-5

**Published:** 2026-04-09

**Authors:** Duygu Kurtulus, Oguz Gurler

**Affiliations:** 1 Department of Physical Medicine and Rehabilitation, Faculty of Medicine, Uskudar University, Memorial Ataşehir Hospital, Istanbul, Turkey; 2 Department of Rheumatology, Faculty of Medicine, Medical Park Samsun Hospital, Istinye University, Samsun, Turkey

**Keywords:** Ankylosing spondylitis, Exercise adherence, Patient education, Non-pharmacological treatment, Spinal mobility

## Abstract

**Objective:**

This study aimed to investigate the effects of individualized patient education and structured follow-up on exercise adherence and functional outcomes in biologic-naïve patients with ankylosing spondylitis (AS).

**Methods:**

In this prospective randomized pilot study, 40 AS patients meeting the modified New York criteria were enrolled. Participants were randomized into an intervention group (*n* = 20) receiving individualized education based on the National Ankylosing Spondylitis Society (NASS) booklet with monthly follow-up visits, or a control group (*n* = 20) evaluated only at baseline and month 6. Patients on biologic therapies were excluded. All patients received a standardized home-based exercise program. Clinical outcomes included spinal mobility (BASMI, fingertip-to-floor distance, occiput-to-wall distance), functional status (BASFI, Dougados Functional Index), disease activity (BASDAI), and pain (VAS). Exercise adherence was tracked for six months using patient logs.

**Results:**

Thirty-two patients completed the study (17 intervention, 15 control). The intervention group demonstrated significant within-group improvements in fingertip-to-floor distance (*p* = 0.005), lateral flexion (*p* = 0.040), occiput-to-wall distance (*p* = 0.042), BASMI (*p* = 0.004), and Dougados Functional Index (*p* = 0.021). Exercise adherence was maintained in the intervention group (*p* = 0.407) but declined sharply in controls (*p* < 0.001). Between-group differences in change scores were not statistically significant.

**Conclusion:**

Regular patient education and structured follow-up may improve exercise adherence and functional outcomes in biologic-naïve AS patients. Integrating such approaches into routine care could strengthen non-pharmacological management. This study is one of the first randomized pilot studies in AS to focus on biologic-naïve patients, isolating the impact of educational and behavioral strategies independent of pharmacologic therapy. The blended model of individualized education with monthly reinforcement may offer a scalable, low-cost framework for enhancing adherence in clinical practice.

**Supplementary Information:**

The online version contains supplementary material available at https://doi.org/10.1007/s11845-026-04364-5.

## Key Summary Points

This pilot randomized study evaluated whether individualized patient education combined with structured follow-up improves exercise adherence in biologic-naïve patients with ankylosing spondylitis.

Patients receiving education and monthly follow-up maintained significantly higher exercise adherence over six months, while adherence declined markedly in the control group.

The intervention group demonstrated significant within-group improvements in spinal mobility and functional outcomes despite the absence of biologic therapy.

Education-based, low-cost interventions may represent a feasible strategy to strengthen non-pharmacological management in routine clinical practice.

**Supplementary Information:**

The online version contains supplementary material available at https://doi.org/10.1007/s11845-026-04364-5.

## Introduction

Ankylosing spondylitis (AS) is a chronic inflammatory rheumatic disease characterized by pain, stiffness, and progressive limitation of spinal mobility [1, 2]. Early diagnosis and comprehensive management are crucial for preventing irreversible structural damage and preserving functional independence [3]. In addition to pharmacological treatment, non-pharmacological interventions—particularly exercise—are strongly recommended by international guidelines [4]. Regular physical activity has been shown to improve spinal flexibility, alleviate pain, reduce fatigue, and enhance quality of life in patients with AS [5].

Despite these benefits, the long-term effectiveness of exercise depends heavily on sustained adherence, which is frequently suboptimal in real-world practice. Barriers such as insufficient knowledge, low motivation, psychological factors, and lack of structured follow-up contribute to poor compliance [6]. Patient education and regular support are promising strategies to address these challenges. Previous studies suggest that education, especially when combined with systematic follow-up, can improve adherence and self-management in chronic musculoskeletal diseases [7]. However, most available data focus on supervised exercise programs or pharmacologically treated patients. Evidence is limited regarding the impact of individualized education and structured follow-up in biologic-naïve AS patients, a group in which low-cost, scalable interventions are particularly relevant.

The aim of this study was therefore to evaluate the effects of individualized patient education combined with monthly structured follow-up on exercise adherence (primary outcome) and on functional and clinical outcomes (secondary outcomes) in biologic-naïve patients with AS. By excluding patients on biologic therapies, this study sought to isolate the contribution of educational and behavioral strategies independent of potent anti-inflammatory treatment, thereby filling a gap in the current literature.

## Materials and methods

This was a single-center, prospective, randomized controlled pilot study conducted between December 2024 and June 2025 at the Physical Medicine and Rehabilitation Clinic of Ümraniye Training and Research Hospital. The protocol was approved by the institutional ethics committee (Approval No: E-54022451-050.05.04-58502), and all participants provided written informed consent. The study was reported in accordance with the CONSORT 2010 statement.

A total of 40 patients diagnosed with ankylosing spondylitis (AS) according to the modified New York criteria were enrolled. Eligible participants were aged 20–60 years, had a stable clinical condition, and were able to perform home-based exercises. Exclusion criteria included pregnancy, claustrophobia, major systemic comorbidities, and the use of biologic therapies (including IL-17 or JAK inhibitors) to eliminate pharmacological confounding.

**Fig. 1:**
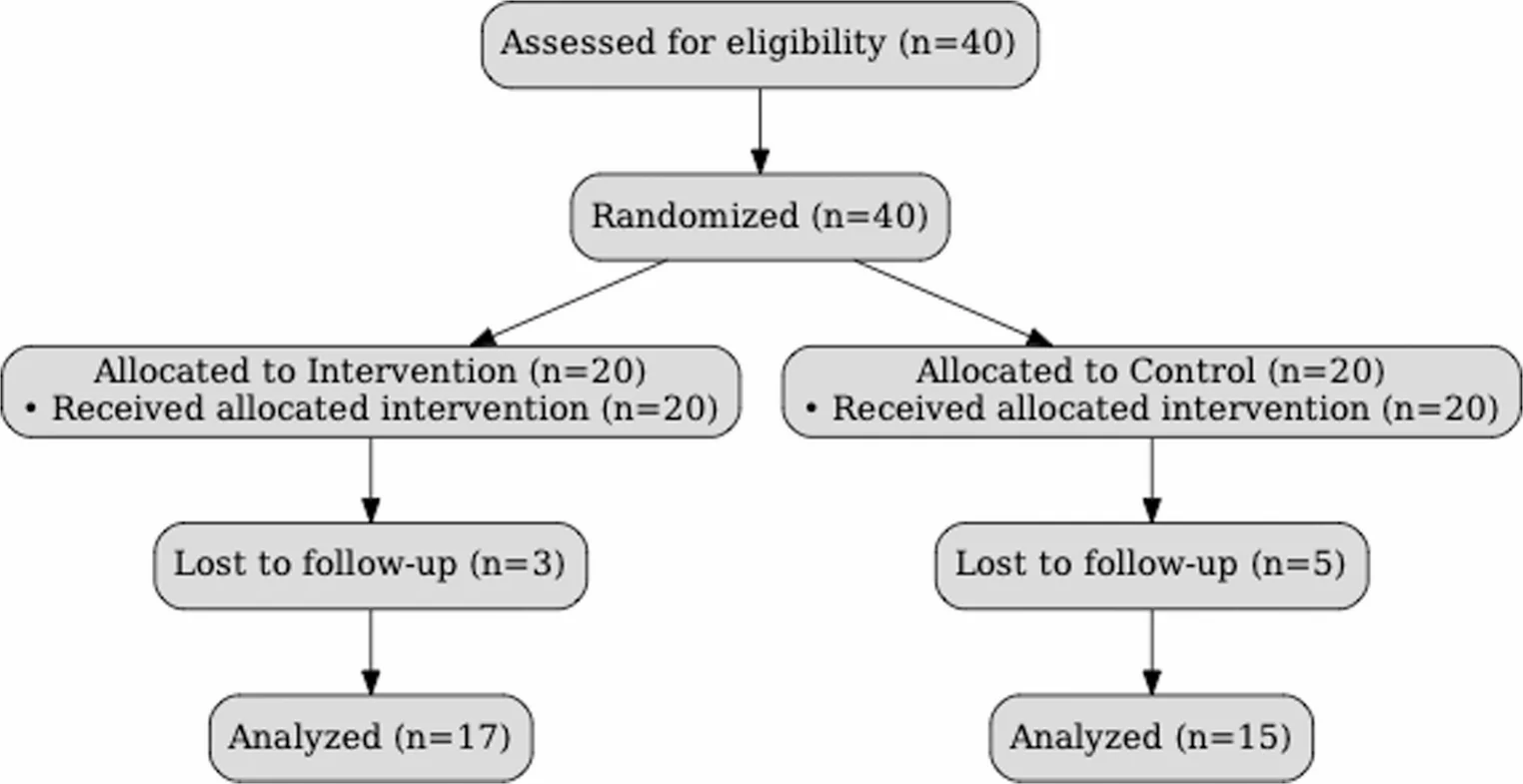
CONSORT flow diagram of patient recruitment, allocation, follow-up, and analysis

Participants were randomly assigned (1:1) into an intervention group (*n* = 20) or a control group (*n* = 20) using a computer-generated sequence prepared by an independent researcher. Allocation concealment was ensured through sealed opaque envelopes. Due to the nature of the intervention, patients and treating clinicians could not be blinded; however, outcome assessors were blinded to group allocation. The patient flow through the study is summarized in Fig. 1.

All participants received a standardized home-based exercise program, consisting of daily exercises with 10 repetitions each. The intervention group additionally received individualized one-on-one education based on the National Ankylosing Spondylitis Society (NASS) booklet, delivered by a physician and physiotherapist, and attended monthly follow-up visits where exercise logs were reviewed. The control group received only baseline and 6-month evaluations and returned exercise logs at the final visit.

The primary outcome was exercise adherence, defined as the percentage of prescribed exercises completed over six months according to patient logs. Secondary outcomes included spinal mobility (Bath Ankylosing Spondylitis Metrology Index [BASMI] [8], fingertip-to-floor distance, occiput-to-wall distance, chest expansion, thoracolumbar lateral flexion, cervical rotation, Schober test), functional status (Bath Ankylosing Spondylitis Functional Index [BASFI] [9], Dougados Functional Index [DFI] [10]), disease activity (Bath Ankylosing Spondylitis Disease Activity Index [BASDAI] [11]), and pain (Visual Analog Scale [VAS] [12]).

As a pilot study, no formal sample size calculation was performed a priori. A total of 40 participants was targeted to provide feasibility and preliminary estimates. Post-hoc analysis indicated that with 32 completers, the study achieved approximately 70% power to detect a moderate effect size (Cohen’s d ≈ 0.6) in adherence outcomes at α = 0.05. These data will inform sample size requirements for future large-scale trials.

### Statistical analysis

All statistical analyses were performed using IBM SPSS Statistics version 25.0 (IBM Corp., Armonk, NY, USA). Descriptive statistics were presented as mean ± standard deviation for continuous variables and as number and percentage for categorical variables. The Shapiro–Wilk test was used to assess the normality of data distribution.

Between-group comparisons for continuous variables were conducted using the Mann–Whitney U test due to non-normal data distribution. The Wilcoxon signed-rank test was used to evaluate within-group changes from baseline to post-treatment. The Friedman test was employed to assess changes over multiple time points within groups (e.g., exercise adherence). Bonferroni correction was applied for pairwise comparisons where appropriate.

A p-value < 0.05 was considered statistically significant.

## Results

A total of 32 patients completed the study and were included in the final analysis (17 in the intervention group and 15 in the control group). Three patients in the intervention group and five in the control group were excluded due to loss to follow-up.

There were no statistically significant differences between the two groups at baseline regarding age, sex distribution, height, weight, education level, occupation, symptom duration, or diagnosis delay (*p* > 0.05 for all). Baseline demographic and clinical characteristics are presented in Table 1.

**Table 1:** Baseline Demographic and Clinical Characteristics of the Study Groups

Variable	Intervention Group (*n* = 17)	Control Group (*n* = 15)	*p*-value
Age (years)	41.59	35.47	0.056
Height (cm)	166.0	168.07	0.583
Weight (kg)	68.47	68.87	0.925
Symptom duration (months)	139.35	112.2	0.427
Diagnosis delay (months)	74.59	63.6	0.880
Education duration (years)	10.07	11.07	0.698
Female (%)	41.2%	40.0%	–

Data are presented as mean ± standard deviation unless otherwise indicated. Mann-Whitney U test was used for comparison

At baseline, clinical parameters including fingertip-to-floor distance, Schober test, chest expansion, thoracolumbar lateral flexion, occiput-to-wall distance, VAS, BASFI, DFI, BASDAI, and BASMI were comparable between groups (*p* > 0.05 for all).

At six months, the intervention group demonstrated statistically significant improvements in fingertip-to-floor distance (*p* = 0.005), lateral flexion (*p* = 0.040), occiput-to-wall distance (*p* = 0.042), Dougados Functional Index (*p* = 0.021), and BASMI score (*p* = 0.004).

No significant improvements were observed in Schober test, chest expansion, VAS, BASFI, or BASDAI scores within the intervention group. In the control group, thoracolumbar lateral flexion (*p* = 0.007) and occiput-to-wall distance (*p* = 0.042) showed statistically significant improvement, whereas all other outcome measures remained unchanged.

Between-group comparisons of change scores for clinical parameters did not show statistically significant differences (*p* > 0.05 for all), as shown in Table 2.

**Table 2:** Pre- and Post-Treatment Change Scores in Key Clinical Outcomes

Outcome Measure	Intervention Group (Δ)	*p*-value (Intervention)	Control Group (Δ)	*p*-value (Control)
Fingertip-to-floor distance (cm)	−5.44	0.005	−1.40	0.176
Lateral flexion (cm)	+ 1.45	0.040	+ 2.80	0.007
Occiput-to-wall distance (cm)	−0.91	0.042	−1.67	0.042
DFI	−3.91	0.021	−2.40	0.099
BASMI	−0.35	0.004	−0.40	0.084
Exercise adherence (%)	+ 4.1	0.407	−35.7	< 0.001

Indicates the change from baseline to month 6. Negative values reflect improvement where lower scores indicate better outcomes (e.g., DFI, BASMI). Statistical significance assessed by Friedman and Wilcoxon tests as appropriate

Regarding exercise adherence, the intervention group maintained a high and stable adherence rate between the 1st and 6th months (82.1% to 86.2%, *p* = 0.407), while the control group demonstrated a marked decline (from 57.8% to 22.1%, *p* < 0.001). Between-group differences in adherence became statistically significant starting from the second month and persisted throughout the follow-up (*p* < 0.01 from month 2 to month 6).

## Discussion

This prospective randomized pilot study evaluated the impact of regular patient education and structured follow-up on exercise adherence and functional outcomes in patients with ankylosing spondylitis (AS). The findings revealed that patients in the intervention group demonstrated improvements in spinal mobility and functional parameters—particularly in fingertip-to-floor distance, lateral flexion, occiput-to-wall distance, Dougados Functional Index (DFI), and BASMI scores—while maintaining high exercise adherence over six months, compared with the declining adherence observed in the control group [13].

Exercise is considered a fundamental non-pharmacological component in the management of AS, with strong recommendations in current clinical guidelines [3, 14]. Regular physical activity improves spinal mobility, reduces stiffness and pain, and helps preserve functional independence over time [5, 15, 16]. However, exercise effectiveness relies heavily on long-term adherence, which is often suboptimal due to motivational, psychological, or educational barriers [6, 17, 18].

Our results support the role of structured patient education in addressing these barriers. The educational intervention, based on the National Ankylosing Spondylitis Society (NASS) booklet and delivered one-on-one by a physician and physiotherapist, allowed personalized guidance on exercise planning and self-monitoring. Similar findings were reported by Tennant et al., who demonstrated that tailored educational support enhances adherence in chronic musculoskeletal diseases [19, 20]. Brophy et al. also reported improved physical performance and adherence following combined education and behavioral support in inflammatory arthritis [7, 21].

A novel aspect of our study is the sustained adherence over six months, suggesting that regular follow-up and engagement are key to maintaining exercise behavior. This aligns with the transtheoretical model’s emphasis on reinforcement, feedback, and reassessment in behavior change [22–24]. TTM-based interventions in other chronic diseases (e.g., osteoarthritis) also showed improvements in long-term exercise adherence [25, 26].

Although within-group improvements were detected in the intervention arm, between-group differences in change scores were not statistically significant, likely due to the limited sample size reducing statistical power [27]. Therefore, the findings should be interpreted with caution, particularly given the limited statistical power of this pilot study. Control group improvements in lateral flexion and occiput-to-wall distance may reflect non-specific effects such as placebo or natural disease variance [28].

Importantly, patients on biologic therapies (IL-17 or JAK inhibitors) were excluded to isolate the impact of educational strategies. This enhances internal validity but limits generalizability to biologic-treated populations.

 This study has several limitations, including the small sample size and single-center design, which limit the generalizability of the findings. Therefore, the results should be interpreted as preliminary and hypothesis-generating. In addition, exercise adherence was assessed using self-reported patient logs, which may be subject to reporting and recall bias. The lack of psychological assessments such as depression or health beliefs is another limitation [29, 30].

Despite limitations, our findings add to the literature on patient-centered education and multidisciplinary management in AS. Structured educational interventions appear feasible, low-cost, and scalable, especially in settings with limited physiotherapy access, and may enhance treatment outcomes.

This study has several notable strengths. First, it focuses exclusively on biologic-naïve patients with ankylosing spondylitis, a group often underrepresented in clinical research. By excluding patients treated with biologic agents, we were able to isolate the effects of individualized education and structured follow-up on exercise adherence and functional outcomes without the confounding influence of potent pharmacologic therapies. To our knowledge, this is one of the first randomized controlled pilot studies to evaluate education-driven adherence strategies in this specific population.

Second, the study introduces a practical and scalable model of patient education by combining standardized written material (the NASS booklet) with personalized physician- and physiotherapist-led sessions and monthly follow-up visits. This blended approach integrates evidence-based educational resources with continuous reinforcement and monitoring, enabling sustained exercise adherence over time. The design also demonstrates how relatively low-cost and accessible interventions can be embedded into routine clinical practice, providing a novel framework for non-pharmacological management of ankylosing spondylitis that can be adapted to diverse healthcare settings.

## Conclusion

This study suggests that regular patient education combined with structured follow-up may enhance exercise adherence and may contribute to improvements in spinal mobility and functional outcomes in patients with ankylosing spondylitis. Excluding patients on biologic therapy allowed a clearer assessment of the educational intervention’s effectiveness. These findings underscore the importance of integrating individualized education and monitoring strategies into routine care for biologic-naïve AS patients. Further large-scale studies are warranted to validate these results and explore long-term sustainability.

## Supplementary Information

Below is the link to the electronic supplementary material.

Supplementary Material 1.

Supplementary Material 2.

Supplementary Material 3.

## Data Availability

The datasets generated and/or analyzed during the current study are available from the corresponding author upon reasonable request.

## Abbreviations

AS: Ankylosing spondylitis
BASDAI: Bath Ankylosing Spondylitis Disease Activity Index
BASFI: Bath Ankylosing Spondylitis Functional Index
BASMI: Bath Ankylosing Spondylitis Metrology Index
VAS: Visual Analog Scale
DFI: Dougados Functional Index
NASS: National Ankylosing Spondylitis Society
JAK: Janus kinase
